# Cost-Effective Encryption-Based Autonomous Routing Protocol for Efficient and Secure Wireless Sensor Networks

**DOI:** 10.3390/s16040460

**Published:** 2016-03-31

**Authors:** Kashif Saleem, Abdelouahid Derhab, Mehmet A. Orgun, Jalal Al-Muhtadi, Joel J. P. C. Rodrigues, Mohammed Sayim Khalil, Adel Ali Ahmed

**Affiliations:** 1 Center of Excellence in Information Assurance (CoEIA), King Saud University, Riyadh 12372, Saudi Arabia; abderhab@ksu.edu.sa (A.D.); jalal@ccis.edu.sa (J.A.-M.); joeljr@ieee.org (J.J.P.C.R.); sayimkhalil@ksu.edu.sa (M.S.K.); 2 Intelligent Systems Group (ISG), Department of Computing, Macquarie University, Sydney, NSW 2109, Australia; mehmet.orgun@mq.edu.au; 3 Faculty of Information Technology, Macau University of Science and Technology, Avenida Wai Long, Macau 999078, China; 4 College of Computer and Information Sciences (CCIS), King Saud University, Riyadh 12372, Saudi Arabia; 5 Instituto de Telecomunicações, University of Beira Interior, Marquês d’Ávila e Bolama, Covilhã 6201-001, Portugal; 6 University of Fortaleza (UNIFOR), Fortaleza-CE 60115-170, Brazil; 7 Information Technology, FCITR, King Abdulaziz University, Rabigh 21911, Saudi Arabia; engadel2003@hotmail.com

**Keywords:** autonomous, communication, cost-effective, cryptography, efficient, key management, routing protocol, secure, wireless sensor networks

## Abstract

The deployment of intelligent remote surveillance systems depends on wireless sensor networks (WSNs) composed of various miniature resource-constrained wireless sensor nodes. The development of routing protocols for WSNs is a major challenge because of their severe resource constraints, *ad hoc* topology and dynamic nature. Among those proposed routing protocols, the biology-inspired self-organized secure autonomous routing protocol (BIOSARP) involves an artificial immune system (AIS) that requires a certain amount of time to build up knowledge of neighboring nodes. The AIS algorithm uses this knowledge to distinguish between self and non-self neighboring nodes. The knowledge-building phase is a critical period in the WSN lifespan and requires active security measures. This paper proposes an enhanced BIOSARP (E-BIOSARP) that incorporates a random key encryption mechanism in a cost-effective manner to provide active security measures in WSNs. A detailed description of E-BIOSARP is presented, followed by an extensive security and performance analysis to demonstrate its efficiency. A scenario with E-BIOSARP is implemented in network simulator 2 (ns-2) and is populated with malicious nodes for analysis. Furthermore, E-BIOSARP is compared with state-of-the-art secure routing protocols in terms of processing time, delivery ratio, energy consumption, and packet overhead. The findings show that the proposed mechanism can efficiently protect WSNs from selective forwarding, brute-force or exhaustive key search, spoofing, eavesdropping, replaying or altering of routing information, cloning, acknowledgment spoofing, HELLO flood attacks, and Sybil attacks.

## 1. Introduction

**Figure 1:**
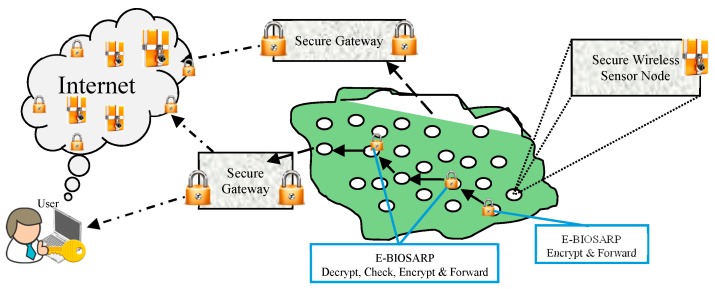
Illustration of a secure WSNs.

In wireless communication, low-cost miniaturized devices that can be used for monitoring in a number of diverse applications have been introduced [1]. These devices are equipped with one or more sensors to detect, for example, humidity, light, sound, or temperature, and are called wireless sensor nodes. Wireless sensor nodes can also communicate with each other in an autonomous manner. Multiple wireless sensor nodes—which are extremely small and have limited battery power, communication bandwidth, and storage space—can be deployed in a particular region to form a wireless sensor network (WSN) in an *ad hoc* manner. The wireless sensor nodes fetch the required data and transfer it hop by hop securely from the source to a single or multiple destinations [2], as shown in Figure 1. In a WSN, the destination (e.g., the sink node or gateway) can be either single or multiple and either static or mobile depending on the requirements of the application scenario.

Routing strategies and WSN modeling have recently received a great deal of attention. The security issues associated with WSNs require strong attack-repelling mechanisms coupled with subsequent verification [3,4,5]. Numerous security strategies are available, such as DTRAB [6] and ReTrust [7], as well as other types of communication mechanisms and solutions that also include single-hop communication as proposed in [8,9,10,11]. However, these strategies are completely infeasible and inapplicable in the case of large-scale WSNs. Many of the data-routing protocols used in WSNs are simple, making them more vulnerable to widespread attacks [12]. In such a WSN, an adversary can deploy his own node(s) to generate several types of attacks for denial of service (DOS) or to compromise existing nodes. Through these compromised nodes, the adversary can perform network-layer attacks, which predominantly involve the manipulation of important data. Attacks that attempt to manipulate data can be categorized into two classes: in the first, the attacker attempts to influence the user data directly, and in the second, the attacker attempts to affect the core data-routing topology.

It is important to focus on the security requirements throughout the initiation and implementation of a WSN design [13]. In the complete lifespan of a WSN, the initialization phase is critical and requires efficient and active security measures. When nodes are deployed, they must acquire and/or build information regarding their neighboring nodes and the environment to enable them to communicate and transfer data to the destination [14]. Communications in a WSN are vulnerable to massive attacks, particularly when the wireless sensor nodes are deployed in hostile environments, because sensor nodes are limited in terms of resources such as battery power, processing speed, signal strength, and storage space. The possible types of attacks on WSN communications include spoofing, tampering, eavesdropping and signal jamming, replaying or altering routing information, wormhole attacks, resource exhaustion, selective forwarding, Sybil attacks, flooding attacks, sinkhole attacks, and even unknown attacks [15,16,17]. Researchers have proposed mechanisms that are mostly dependent on secure authentication and encryption [18] to thwart attacks, but data confidentiality remains a challenge [4,19].

In many aspects of technology, nature has provided inspiration for many solutions. For example, Eigen, Schuster and Von Foerster [20,21] proposed the application of biological self-organization methods to network data using primary engineering applications [22]. Communications technology has also heavily benefitted from biologically inspired mechanisms; for example, a BIOlogy-Inspired Self-organized Secure Autonomous Routing Protocol (BIOSARP) [23,24,25] has been developed based on an artificial immune system (AIS) that begins by checking the behavior of neighboring nodes and classifying them once the neighbor table of each sensor node is populated with the relevant information. However, before the AIS-based preventive measure is initiated, adversaries have an open opportunity to seize control of the entire deployed network. We have found that BIOSARP is adequate in terms of cost, but is weak in all aspects of providing security. By contrast, Secure Real-Time Load Distribution (SRTLD) is adequate in terms of security but not cost. Thus, by combining the best features of SRTLD and BIOSARP in a novel way, we have developed a secure and cost-efficient solution.

This paper proposes an enhanced BIOSARP (E-BIOSARP) and presents a detailed description of its architecture, a thorough analysis, and a comprehensive comparison with other schemes to demonstrate its efficiency. E-BIOSARP incorporates a random key encryption and decryption mechanism [26] into BIOSARP to provide active security measures for WSNs. Saleem *et al.* [27] presented the initial design of and preliminary results regarding E-BIOSARP. Because of the encryption mechanism used in E-BIOSARP, WSN communication is secured even during the critical learning and initialization phases. An extensive security and performance analysis was conducted to demonstrate the efficiency of E-BIOSARP. The main contributions of this paper are as follows:

- By incorporating a random key encryption and decryption mechanism [26] into BIOSARP, we propose an enhanced BIOSARP named E-BIOSARP to provide overall network communication security from the very beginning of the establishment of a WSN at a low performance cost.
- Our security analysis shows that E-BIOSARP can ensure confidentiality and integrity by encrypting header fields such as the packet sequence ID (*Pkt_ID*), destination ID (*D_ID*), and source ID (*S_ID*) and by transmitting fragmented data instead of applying cryptographic operations to complete packets, as is the case in most security mechanisms.
- Our security analysis also shows that E-BIOSARP is resilient against data tampering attacks, selective forwarding, brute-force or exhaustive key search, spoofing, eavesdropping, replaying or altering of routing information, cloning, acknowledgment spoofing, HELLO flood attacks, and Sybil attacks.
- We present the implementation of a WSN scenario using E-BIOSARP in network simulator 2 (ns-2) and the population of this scenario with malicious nodes to analyze the behavior and performance of E-BIOSARP.
- E-BIOSARP is analytically and experimentally compared in terms of processing time with state-of-the-art security mechanisms, including TinyHash, TinySec with authenticated encryption (TinySec-AE), TinySec with authentication only (TinySec-Auth), exclusion basis systems (EBSs), location-based resilient security with authentication (LBRS-Auth), and SRTLD.
- Simulation results show that E-BIOSARP outperforms SRTLD in terms of energy consumption, packet overhead and delivery ratio and also outperforms another state-of-the-art secure routing protocol, the energy-efficient secure path algorithm (ESPA), in terms of energy consumption.

This paper is structured as follows: Section 2 discusses recent related works. Section 3 describes the architecture of E-BIOSARP. Section 4 presents a security analysis of E-BIOSARP. Section 5 provides a complexity analysis of E-BIOSARP and other security mechanisms. Section 6 discusses the simulation-based implementation and the experimental results. Section 7 concludes the paper with a summary of our contributions and a brief discussion of future work.

## 2. Related Work

In a WSN, security must be enabled before the initialization phase, at the time of network deployment, because node cloning attacks can be executed only during this phase, as stated by Lim [28]. Moreover, clustering-based secure routing mechanisms are infeasible for WSNs because of the energy constraints of wireless sensor nodes [29]. Elliptic curve cryptography (ECC) can be implemented on miniaturized wireless sensor nodes, thereby making public key cryptography (PKC) a realistic possibility for these sensors [30,31]. However, the implementation of PKC-based security at higher layers on top of the efficient primitives is not a simple process because of the unique requirements and characteristics of wireless sensor nodes. Consequently, studies of the performance of PKC-based security schemes have been limited in scope.

A secure energy-efficient routing protocol (SERP) for densely deployed WSNs was proposed by Pathan and Hong [32]. This tree-based routing method depends on the distance and the remaining energy parameters of nodes for routing decisions. The authors use one-way hash chains and shared secret keys that are pre-stored in the wireless sensor nodes before deployment. During initialization, all nodes in a WSN are in active mode for the construction of the tree and the one-way hash chain. In SERP, the encryption key changes occasionally, providing a security advantage. However, a major disadvantage of SERP is that a high traffic overhead is generated when the shared key is changed throughout the entire network. Furthermore, the mechanism presented by Pathan and Hong [32] involves a key refreshment system that is called when all nodes on a certain path are offline until the destination is reached, which entails the re-initialization of part of or even the entire network. Because of the involvement of a time parameter, feedback and other processes, such as shared key updating, long processing times are required, which cause delays and consume large amounts of energy. In addition, because the shared secret key—which is not generated by the system in real time and is non-dynamic—is preloaded in the base station and the sensor nodes, the mechanism is unrealistic for application in highly dynamic WSN scenarios. In SERP [32], the source node ID is open and can be easily read by searching packet headers, which opens the system to brute-force search and eavesdropping attacks. The authors conducted a detailed comparison of their proposed SERP with LEACH and EAD in an ns-2-based simulation. LEACH energy consumption is high because it uses one-hop transmission to reach the cluster head or the sink node, and therefore, EAD performs much better than LEACH because of its multi-hop transmission capability. However, by virtue of its adaptive transmission range, SERP consumes less energy than does either LEACH or EAD.

Ahmed and Fisal [26] proposed a secure real-time load distribution (SRTLD) routing protocol to ensure secure packet transfer by means of packet header authentication. SRTLD was developed as an enhancement of the real-time load distribution (RTLD) routing protocol [26] through the incorporation of a security module. The security module contains a mathematical function that is based on a unique random number generator function [26]. A hash function is further utilized to encrypt the source address (*S_ID*), destination address (*D_ID*), and packet ID (*Pkt_ID*) fields in the packet header. When a node receives a packet, it attempts to decrypt the encrypted fields, and thus, the data authenticity in the network is verified by SRTLD. The authors showed that SRTLD is capable of securing WSNs from wormholes, HELLO flood attacks, Sybil attacks, selective forwarding, and sinkholes [26]. The main shortcoming of SRTLD is that each node is programmed with a predefined location and with routing optimization values depending on the target location or territory. Thus, every time the network topology or target area conditions change, the routing optimization and location parameters must be reevaluated, and accordingly, all sensor nodes must be reprogrammed, which is highly impractical. Furthermore, in SRTLD, the authors also assume that the sink node must be a trusted computing base.

Jean de Dieu *et al.* [29] presented ESPA for WSNs with the goal of ensuring the authenticity and integrity of sensed data. The mechanism presented by Jean de Dieu *et al.* [29] depends on shared secret keys and one-way hash functions. The shared key is known by the sensor nodes and the base station. The authors proposed a general ant colony optimization (ACO)-based energy-aware routing (DEAR) algorithm. The routing decision depends on two metrics related to the distance and remaining battery power of the nodes. In presenting their security scheme, the authors assumed that the main commands are generated from a centralized location, that is, the base station or a gateway. The general ACO architecture depends on ACO_forward (ACO_F) travel from the source to the destination and ACO_backward (ACO_B) travel back from the destination to the source. This generates massive traffic overhead and causes the network to consume an enormous amount of energy. In a WSN scenario in which the network contains a large number of wireless sensor nodes that communicate in a multi-hop manner, feedback (ACO_B) is practically impossible. In addition, if the shared key information has not been shared with a particular node, then that wireless sensor node may be prohibited from participating in the network and any communications therein; consequently, the entire network could collapse at any time. Jean de Dieu *et al.* [29] analyzed the performance of ESPA in terms of three defense parameters (anonymity, confidentiality, and integrity) and compared energy-efficient ant-based routing algorithm (EEABR), secure energy-efficient routing protocol (SERP) and energy-efficient secure pattern based data aggregation (ESPDA) using MATLAB software. The authors claimed that ESPA performs better than the SERP and ESPDA methods.

In [33], the authors proposed a scheme called Extended Two-hop Keys Establishment (ETKE) to protect the hop count information without referring to the sink node. Furthermore, the authors proposed a novel secure routing protocol named Secure Multi-pAths Routing for wireless sensor neTworks (SMART) based on the ETKE key management scheme. The authors claimed that SMART is resilient against forged hop count attacks and outperforms SeRINS in terms of energy consumption.

The authors of [34,35] proposed BIOARP, which employs enhanced ant colony optimization (eACO) [36] to select optimal routes from a source node to a destination node in a WSN. BIOARP provides a self-optimized routing mechanism that enables improved data throughput, extends the WSN lifetime and minimizes delays with less data loss. Saleem *et al.* [23] also proposed BIOSARP, an enhanced version of BIOARP, by adding a self-security management module that relies on an AIS. The ability of BIOSARP to detect, classify, and remove non-self nodes is analogous to the human immune system (HIS) because the AIS is a derivation of the HIS in the computer domain. The primary function of the HIS is the detection of abnormalities by distinguishing between self and non-self entities. Analogously, the primary function of BIOSARP is to detect attacks and classify self and non-self nodes.

The major limitation of BIOSARP is that it requires time to develop the knowledge of the overall network during the initialization phase of WSN deployment. The initialization phase is a critical period during the complete lifespan of a WSN and requires efficient and active security measures. When nodes are deployed, they must acquire and/or build information regarding their neighboring nodes and the environment to enable them to communicate and transfer data to the destination. In BIOSARP, the AIS begins to check neighboring node behavior and to execute the classification process only once the neighbor table is populated because the AIS depends on the routing table information. Until the AIS-based preventive measure is initiated, adversaries have an opportunity to seize control of the entire deployed network.

Sookhak *et al.* [18] presented a method that can detect dynamic and static wormhole attacks in geographic routing protocols (DWGRP). DWGRP depends on a pairwise key pre-distribution scheme with the assistance of beacon packets. When a node is initialized, the beacon packets of the new structure are broadcast, and a neighbor table is generated in reply. The authors conducted an ns-2-based simulation to evaluate DWGRP in terms of the probability of missed detection, wormhole detection rate, and resend packet rate. However, DWGRP has several drawbacks. The main drawback is that when the beacon packets are broadcast, DWGRP sends out unencrypted information that can be easily hacked, thus compromising the entire network. Furthermore, in the simulation study, each node updated its neighborhood table every 0.3 s by broadcasting beacon packets. This behavior implies that the entire network will be busy updating neighbor tables essentially at all times; however, this is totally unrealistic and is itself a DDOS attack. In a WSN, numerous broadcasts cause battery life to decrease and collisions to occur, resulting in packet loss. Hence, the underlying concept of DWGRP is not practical, and it is very costly because the nodes require a global positioning system (GPS).

Zhang *et al.* [37] proposed an elliptic curve cryptography-based secure energy-efficient access-control scheme. Their theoretical security analysis showed that the proposed mechanism addresses replay attacks, impersonation attacks, and sensor node spoofing attacks.

In [38], the authors proposed the Double Cluster Heads Model (DCHM) based on clustering, trust systems, and data fusion. This model involves the selection of two cluster heads per cluster to perform data fusion independently. The authors claimed that the proposed algorithm detects and prevents attacks on a WSN based on the assumption that compromised cluster heads will generate random results and the outputs received from the two cluster heads will therefore differ. The proposed concept of a system based on reputation and trust depends on feedback (from the base station to the cluster heads) and information gathering, which generate packet overhead. Furthermore, the authors assumed that the base station is a trusted computing platform and that at the time of programming, every node will be assigned three keys, namely, a master key, a pairwise key and a cluster key. Among these three static keys, the cluster key is unique for each cluster, introducing considerable complexity in the programming and deployment of nodes. In their threat model, the authors considered attacks that mislead the user, namely, jamming, the falsification of local values, and the falsification of fusion results. In their performance evaluation, comparisons between compromised sensor nodes and credible sensor nodes were first conducted, and the DCHM results were then compared with those for BRSN [38] in terms of the security of data fusion, accuracy, and average energy consumption over the network lifetime.

In [39], the authors proposed a scheme called Fully homomorphic Encryption based Secure data Aggregation (FESA) in large-scale wireless sensor networks (LWSNs) based on message authentication codes (MACs) to support arbitrary aggregation operations and provide end-to-end data confidentiality. This scheme involves complete data encryption and incurs increased computational overhead because of its large public key size. Therefore, it is not feasible, particularly for LWSNs. The work reported in [40] also provided a secure data aggregation routing protocol, but unfortunately, it is also based on MACs; in addition, the digital signature and the remaining supporting parameters cause the packets to be very large, making them difficult to process and leading to the consumption of enormous amounts of energy.

The physical and MAC layers of a WSN are based on IEEE standard 802.15.4, which was specifically developed for low-rate data transfer. Table 1 briefly compares the most recent network layer protocols for security in WSNs. The detailed literature review presented above yields the conclusion that secure data routing in WSNs is complicated by WSN resource constraints, such as limitations on power, signal strength, reliability of wireless communication, and memory storage. The shortcomings of the reviewed security mechanisms, as summarized in Table 1, prevent them from successfully securing WSNs against most network-layer attacks. Therefore, in the design of a security mechanism specifically for WSNs, the abovementioned limitations should be considered.

## 3. The Architecture of the Proposed E-BIOSARP

The architecture of E-BIOSARP is based on BIOSARP but is further enhanced with a random generator based on a mathematical function [26], the same as that incorporated into SRTLD. The idea behind E-BIOSARP is that BIOSARP has better performance with respect to throughput with better network life, whereas the encryption mechanism of SRTLD provides communication security in terms of integrity and confidentiality.

**Figure 2:**
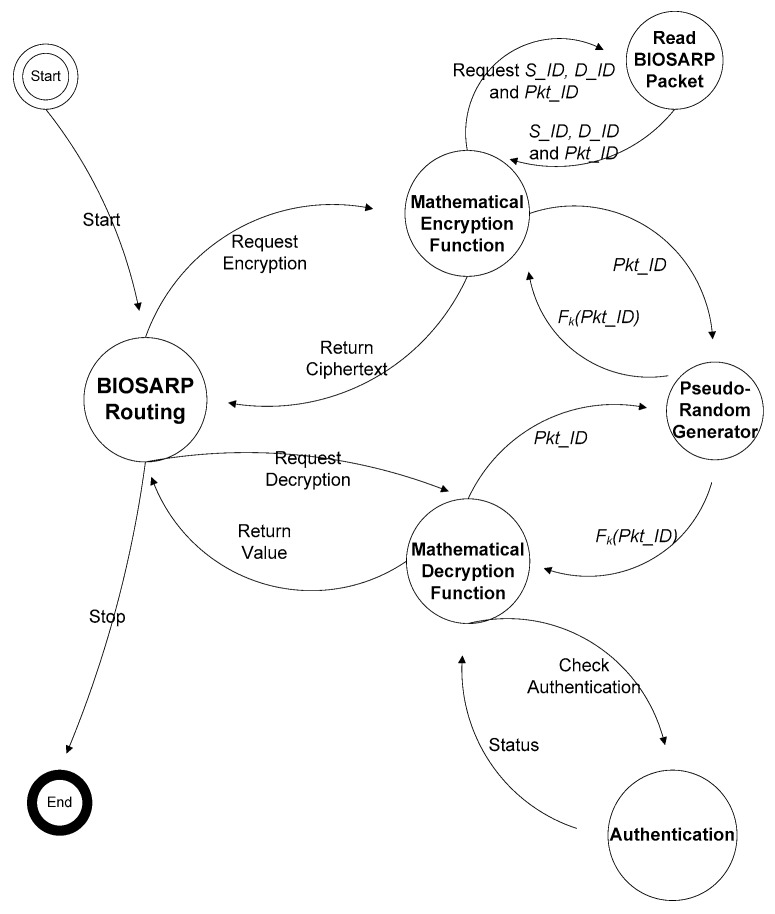
State machine diagram for packet encryption and decryption [27].

Under E-BIOSARP, the workflow of the encryption mechanism begins when a packet is to be forwarded to the next self-node. During forwarding, the BIOSARP routing mechanism, as shown in Figure 2, requests encryption from the Mathematical Encryption Function, which encrypts only some of the fields in the packet header (*Pkt_ID*, *D_ID*, and *S_ID*) rather than the payload or the complete packet, which has already been fragmented. The structures of the ready-to-receive (RTR) packet and the data packet header in E-BIOSARP are presented in [35]. As a result of encryption, if malicious nodes and/or adversaries capture a secure packet, they cannot read the packet’s movement or its destination, which in turn ensures the privacy of its origin. The following assumptions were made while developing the encryption mechanism, which is based on a random key approach and dynamic mathematical computations:

- Two master keys (*k1* and *k*) generated with a pseudo-random function are injected into each sensor node while it is flashing its read-only memory (ROM). Here, *k1* is a master key for the new node, and *k* is a master key for all nodes.
- Before deployment, every node is set with a hard mathematical function and its reverse computation.

The flow diagram of the encryption and decryption process is presented in [27]. In this paper, the state machine diagram is presented in Figure 2 and is explained in detail to elucidate every step and process at the hardware level. Whether the data arrive from the application layer or a data packet is received from a neighboring node, the BIOSARP routing state is called. If the data to be forwarded arrive from the application layer, then the E-BIOSARP routing mechanism sends a request to the Mathematical Encryption Function for encryption. The mathematical function reads the packet details and acquires the values of *Pkt_ID*, *D_ID* and *S_ID*. *D_ID* and *S_ID* are encrypted by the mathematical function based on *k* and *Pkt_ID*. The encrypted packet, with hidden *D_ID* and *S_ID* values, is sent to the MAC layer, as shown in Figure 2, for the frame to be forwarded to the optimal neighboring node. The encryption of all data in a packet is avoided because this process would consume an enormous amount of energy and because the information is split into a number of data packets (because the packet size in a WSN is very small). Moreover, because of the encoding of the packet sequence ID, source ID and destination ID, the data packets become fully secure because they cannot be processed by adversaries and thus are unrecognizable to them.

When a data packet is received from the MAC layer, the BIOSARP routing mechanism sends a request to the mathematical function for decryption, as shown in Figure 2. In the decryption process, the algorithm decrypts the destination ID (*D_ID*) and source ID (*S_ID*). Because every self-node in the network contains a random generator function, each node can generate the same random key based on the values of *Pkt_ID* and *k* to decrypt the header fields of the packet. The decryption function performs authentication by evaluating the output. The status is marked as normal by the authentication state if the output is between 0 and *R*, and as a result, the mathematical decryption function is returned to the BIOSARP routing mechanism to process the packet. Furthermore, in the case that a new wireless node wishes to join the network, a control packet is transmitted that contains the newly encrypted *R* with the master key *k1*.

**Figure 3:**
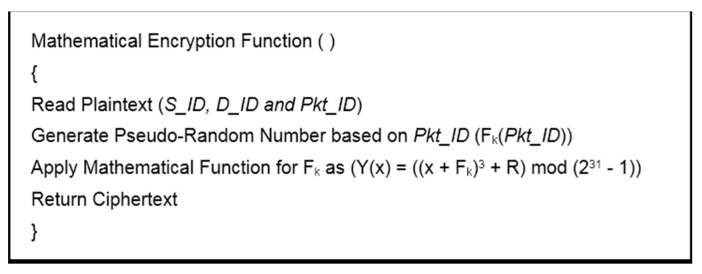
Encryption with authentication.

**Figure 4:**
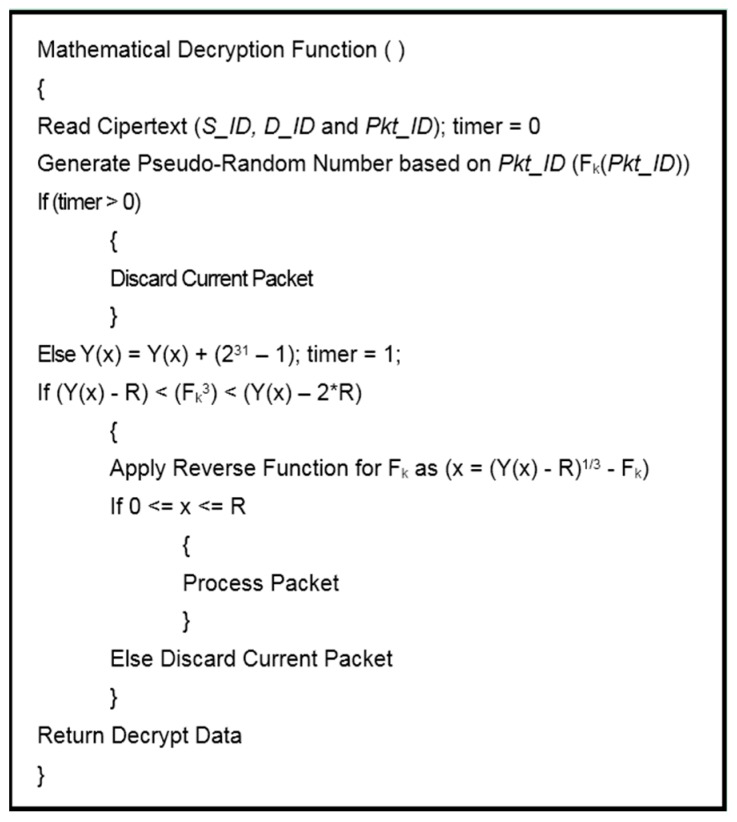
Decryption with authentication.

In BIOSARP, the AIS-based autonomous security mechanism requires a certain amount of time after deployment to accrue the necessary knowledge and to begin classification [23,24,25]. A WSN in this initialization phase is insecure and, as a result, can be hacked, taken over or damaged by adversaries. Therefore, E-BIOSARP is enhanced with packet encryption to secure the WSN during the initialization stage. During communication over a WSN, the proposed mechanism encrypts and decrypts the control and data packets. The algorithm pseudo codes for encryption with authentication and decryption with authentication are presented in Figure 3 and Figure 4, respectively. Both processes begin with the reading of the packet header fields *D_ID* and *S_ID*. The node will further process the packet if it is authenticated; otherwise, the node drops/discards the packet.

During the encryption process, the *Pkt_ID*, *D_ID*, and *S_ID* values are obtained from the header of the packet that has just been received. Of the extracted values, *Pkt_ID* and *k* are utilized to generate a pseudo-random number. The Mathematical Encryption Function is then used to encrypt the packet header fields before the secure packet is sent. The maximum field size is 4 bytes (2^31^ − 1), and if the encryption function generates a number greater than this maximum size, the modulo function will be used, as shown in Figure 3.

At the other end of the transmission process, when a node receives a packet with encrypted header fields, it calls the Mathematical Decryption Function to perform the decryption based on the *Pkt_ID*, *D_ID*, and *S_ID* values extracted from the received packet header and the pseudo-random number calculated using *Pkt_ID* and *k*, as shown in Figure 4. The *D_ID* and *S_ID* values are verified using the modulo function (2^31^ − 1) in reverse to ensure the authenticity of the packet. If the value obtained in this reverse process is between *0* and *R*, then the packet is categorized as legitimate and is further processed; otherwise, it is discarded.

## 4. Security Analysis

This section presents a security analysis of our proposed E-BIOSARP system. We utilize the security analysis method presented in Wang *et al.* [30]. To simplify the analysis, we focus on the critical components of our model, including packet elements and secure transmission. We begin the analysis from the discovery phase, which involves the secure transfer of information between self-nodes to form a private community or to communicate with a new node.

**Figure 5:**
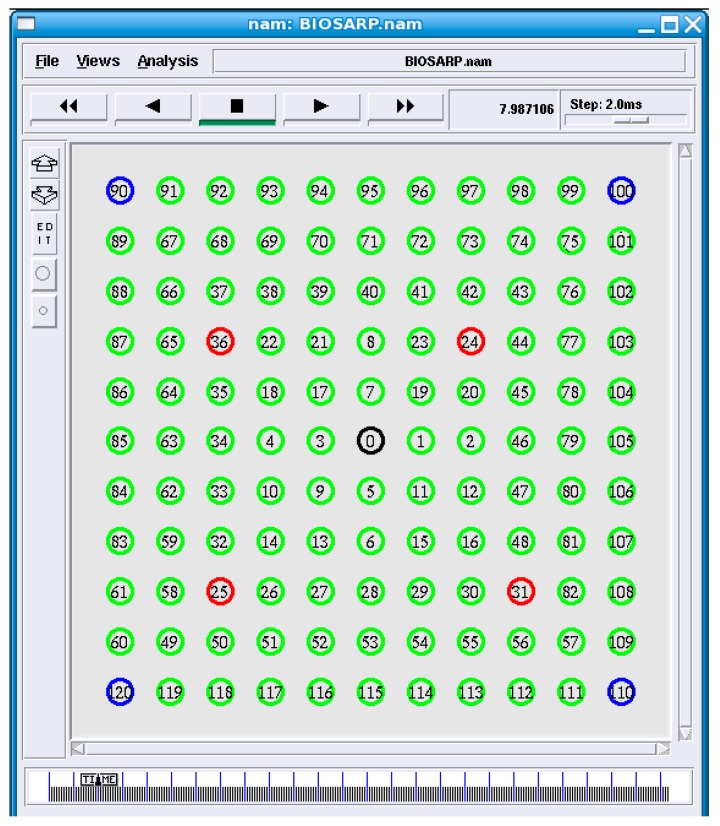
Network simulation grid (source nodes (blue), sensor nodes (green), malicious nodes (red), and sink node (black)).

When a node broadcasts a route replay request packet, it encrypts the important contents, which can only be decrypted by nodes that can understand the received packets, that is, only self-nodes. These neighboring self-nodes will then replay their encrypted information to the node that broadcasted the packet. In this manner, a secure private community is constructed to monitor the transmission. The monitored encrypted data are then transferred hop by hop to their destination, namely, the sink node, as shown in Figure 5.

E-BIOSARP combines BIOSARP with the encryption mechanism of SRTLD because BIOSARP offers better packet transfer behavior and the SRTLD encryption mechanism provides better communication security with minimal processing time. Under E-BIOSARP, the packet header fields *(Pkt_ID*, *D_ID*, and *S_ID*) are encrypted instead of the payload or the complete packet, which has already been fragmented. As a result, if malicious nodes and/or adversaries capture a secure packet, they cannot read the packet’s movement or its destination, thereby ensuring the integrity and confidentiality of the network.

The encryption process is based on a function that verifies the integrity of a message via sender identification and ensures that the data are received exactly as sent. The encryption algorithm is based on the maximum value of a 32-bit signed integer, the prime number equal to 2^31^ − 1. The plaintext in important header fields is encrypted and converted to cipher text at every hop. Therefore, the use of this algorithm counters the possible attacks listed below.

- *Attack based on packet dropping*: The proposed encryption mechanism rapidly identifies and only replies to and/or forwards data packets to self-nodes. The authentication process is fast because of the reduced overhead of the proposed encryption mechanism, as elaborated in Section 3, and thus can efficiently handle packet drop attacks.
- *Redirection with modified hop counts*: Redirections [41] are avoided because of the E-BIOSARP authentication process; moreover, the E-BIOSARP RTR packet, as described in [35], does not contain a *hopcount* field. Thus, the network is secure against this type of attack.
- *Node replication or cloning attack*: This type of attack is initiated when an adversary takes the complete information of a node, replicates/clones a node with the same information, and inserts the clone into the network to execute further attacks. E-BIOSARP protects the network from remote node replication or cloning attacks by rapidly identifying non-self/compromised nodes and thus avoids the receiving or sending of any packet from or to such a node.
- *Brute-force search (or exhaustive key search attack)*: In a brute-force search attack, the adversary attempts to acquire a user ID and password. The proposed encryption mechanism uses a random key (*Pkt_ID* and *k*) that changes at the processing of every packet. Furthermore, E-BIOSARP’s rapid identification process makes it extremely difficult for an attacker to acquire the fragmented data or, if they are acquired, to reconstitute them [29].
- *Eavesdropping attack*: The random-key-based encryption mechanism avoids eavesdropping attacks by encrypting the main contents *S_ID* and *D_ID* of the communication; thus, monitoring is secret, and hijacking of confidential data is impossible.

Other types of attacks that are addressed by the general SRTLD encryption mechanism and thus are also efficiently addressed by E-BIOSARP are listed below.

**Figure 6:**
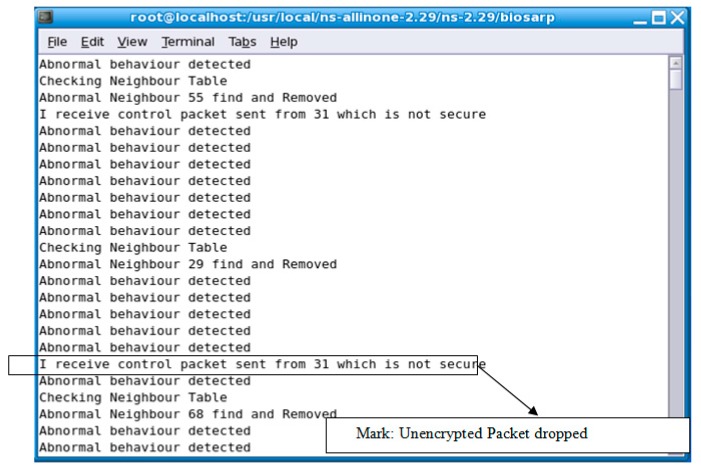
The output of actions taken by E-BIOSARP against adversaries.

- *Altered, spoofed, or replayed routing information attack or wormhole attack*: In this type of attack, the adversary or malicious node targets the routing information exchanged between nodes. The rapid authentication process in E-BIOSARP protects the network from these types of attacks by protecting the packet header. Therefore, when a sensor node transmits a packet, it encrypts the header, and upon receipt, the proposed mechanism confirms that certain fields are encrypted with the correct key. If not, the packet is dropped without being opened, as shown in Figure 6.
- *Acknowledgment spoofing and selective forwarding*: A malicious node executes this type of attack by receiving and dropping packets as they travel through the network; as a result, the nodes at one hop conclude that there is a problem with the forwarding of data packets through the nodes in the routing table. Alternatively, a malicious node may use feedback to alter a node’s decision regarding which strong link to use to send packets. The fast authentication process in E-BIOSARP protects the WSN from acknowledgment spoofing and selective forwarding attacks by rapidly identifying and dropping non-self packets, as shown in Figure 6.
- *Sybil attack*: In a Sybil attack, a malicious node begins advertising itself with multiple IDs. When a self-node receives these RTR packets, they cannot be authenticated because of their different header structure and/or field values, and they are consequently dropped, as presented in Figure 6.
- *HELLO flood attack*: An invader deploys a strong device that generates and broadcasts RTR/HELLO packets with a high signal strength to convince the WSN nodes that the adversary node is a neighboring self-node. Under E-BIOSARP, the possibility of forwarding data to such non-self or HELLO-flood-generated nodes is avoided through the dropping of these false packets.

## 5. Complexity Analysis

In this section, the complexity of E-BIOSARP is studied and compared with those of the security schemes TinyHash, TinySec-AE, TinySec-Auth, EBSS, LBRS-Auth, and SRTLD. The complexity is studied based on the functions involved in each security mechanism by considering the number and processing time of the functions that are executed, which determine the time is required by a node to process a packet. The results of the comparison are presented in Table 2. In the table, we use the following notations: D, P, and H denote the size of a complete data packet, the size of the data payload of a packet, and the header size of a packet, respectively, such that D = P + H. Encrypt(D) and MAC(D) are functions that return the computing times required to apply the Encryption and MAC algorithms, respectively, to information of size D.

In TinySec-AE, a data payload of size P is encrypted using the ciphertext stealing technique [42] to create encrypted data of the same size as the plaintext. TinySec-AE then applies a MAC algorithm to the encrypted data and the packet header, which leads to a computing time of [Encrypt(P’) + MAC(D)]. For TinySec-Auth, this time is reduced to MAC(D) because only the data packet is authenticated with a MAC. The computing time for TinyHash is [Encrypt(D) + MAC(D)] because it uses HMAC for authentication and SHA1 for message digestion. LRBS-Auth and EBSS each create a MAC for the data payload.

Table 2 shows the ranking of the security schemes in terms of the security functions used or complexity. The lowest complexity corresponds to the highest ranking; that is, number “1” represents the highest ranking. The encryption function has a shorter processing time than the MAC algorithm [43]; moreover, (*S_ID* + *D_ID* + *Pkt_ID*) < H < P. Therefore, SRTLD and E-BIOSARP incur the lowest complexity because these approaches need only to encrypt three fields of the packet header, namely, *S_ID*, *D_ID* and *Pkt_ID*.

## 6. Simulation-Based Implementation, Experimental Results and Discussion

In the simulation, the network model was based on IEEE 802.15.4 MAC and physical layers; the network parameters that were used to configure the WSN scenario in ns-2 are given in Table 3. In all simulations, a many-to-one traffic pattern was utilized, and after every 180 s, the routing table record was deleted to keep the neighboring information updated. Similar to the topology used by Ahmed and Fisal [26], the simulation topology consists of 121 nodes distributed in an 80 m × 80 m region, as shown in Figure 5. The nodes numbered 120, 110, 100 and 90 are the source nodes, node 0 is the sink node, and nodes 24, 25, 31 and 36 are the adversary nodes. The performance of E-BIOSARP was evaluated in terms of energy consumption, data packet overhead, and packet delivery ratio.

### 6.1. Simulation of Abnormalities and Attack Countermeasures

Figure 6 shows the countermeasures taken against abnormalities detected in a WSN with the aid of the proposed mechanism. A packet from malicious node 31 is received and dropped, as shown in Figure 6. The encryption/decryption mechanism is the main process that strives to achieve confidentiality, which is the primary requirement for security. Therefore, all sent packets are encrypted, and hence, all received packets must be decrypted. For example, when the sink (or an intermediate node) receives a packet, it will execute the decryption process. If the received packet is decrypted with a legal output, then the sink will consider this packet to be a legal packet originating from a legitimate node. Otherwise, the sink (or intermediate node) will regard this packet as an attack packet. Subsequently, if and when the same node has launched a certain number of attacks at the sink (or intermediate node), then that node will be regarded as an adversary and removed from the neighbor table. Hence, the random-key-based efficient encryption mechanism secures the network from the time of deployment.

### 6.2. Impact of an Increasing Number of Malicious Nodes

**Figure 7:**
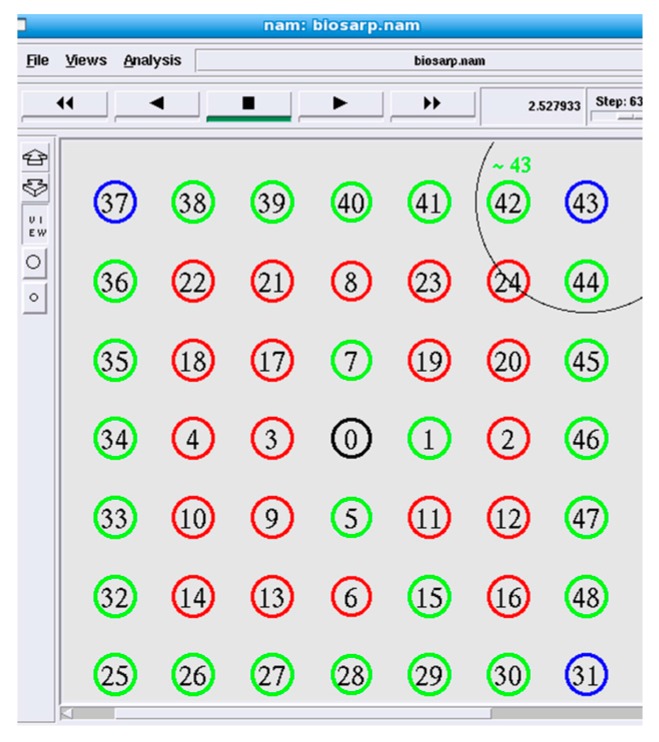
Worst-case scenario with 20 malicious nodes, as indicated in red.

Attacks on the network layer strongly impact the data routing performance. By considering an increasing number of non-self nodes in a WSN, the effect of such attacks is studied in this section. In the simulation, 49 nodes were arranged in a region of 80 m × 80 m, similar to the topology used by Ahmed and Fisal [26]. The packet rate was fixed at 9.6 packets/s, and the number of malicious nodes was varied from 4 to 20. The worst-case scenario with 20 malicious nodes is illustrated in Figure 7.

The performance of E-BIOSARP in terms of data throughput was reasonably maintained, even as the number of malicious nodes increased. E-BIOSARP treats malicious nodes as the hole problem [44]. The hole problem occurs when the nodes in a certain region cannot forward data packets toward their destination. Table 4 shows that as soon as the number of malicious nodes reaches 20, the delivery ratio declines because of communication disruption between the sink and nodes 43, 31 and 37, which are generating the data packets, as demonstrated in Figure 7. At the same time, the energy consumption increases because the transmitting nodes must use considerable power to transmit bits over large distances, as shown in Table 4.

In Table 5, the detection rate and accuracy of E-BIOSARP and SRTLD are compared for the same scenario, as described earlier in this section and shown in Figure 7. The detection rate is defined as the number of illegitimate packets that are detected by the security mechanism at all self-nodes divided by the total number of illegitimate packets received, and this quantity, expressed as a percentage, is the detection accuracy. The detection rate and accuracy are measured as follows:

Detection rate is X/(X + Y), and Accuracy is (X/(X + Y)) × 100%

where:

X is the number of illegitimate packets detected and dropped at all nodes andY is the number of illegitimate packets that are received from adversary nodes and are not detected.

### 6.3. Processing Time Comparison

In Section 5, we analyzed the performance of the security schemes in terms of the processing time required to perform cryptographic operations. The simplest way to monitor this metric is to record the system times immediately before and after the execution of the security mechanism. The problem with this approach is that the system clock in a sensor is not precise. To address this issue, we used the clock of the host PC (*i.e.*, the sink node) and measured another metric equivalent to the processing time, called the security processing time per hop. This metric is defined by Liu *et al.* [45] as follows:

$$\Delta T_{1}=T_{end}^{sec}-T_{start}^{sec}$$

$$\Delta T_{2}=T_{end}-T_{start}$$

where:

$T_{end}^{sec}$: time of packet dispatch from the sending node with the security scheme.
$T_{start}^{sec}$: time of packet reception at the receiving node with the security scheme.
$T_{start}$: time of packet dispatch from the sending node without the security scheme.
$T_{end}$: time of packet reception at the receiving node without the security scheme.

Thus, the time period required for the operating system to execute the security scheme on a node (*i.e.*, the security processing time per hop) is given by the following:

$$\Delta T_{\sec}=\Delta T_{1}-\Delta T_{2}$$

The processing time is a very important parameter in the routing protocol because it affects the overall performance of the network in terms of data throughput and battery power consumption. In [26], the authors showed that compared with LBRS-Auth, TinySec-Auth, EBSS, TinyHash, and TinySec-AE, SRTLD requires the least processing time.

**Figure 8:**
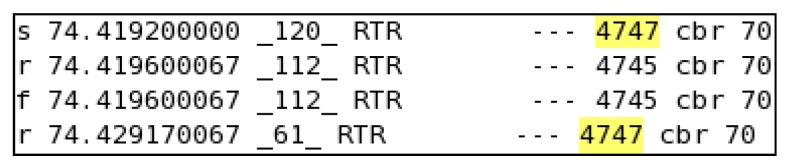
Packet transfer with encryption.

**Figure 9:**
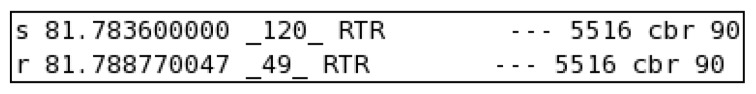
Packet transfer without encryption.

The network topology was configured as in [26] to acquire ns-2-based simulation results. Figure 8 shows the times for the transfer of an encrypted data packet; the transfer time for data packet 4747 was 9.9701 ms, and similarly, from Figure 9, we calculate a transfer time without encryption of 5.17005 ms. The results were generated for both BIOSARP and E-BIOSARP as shown in Figure 8 and Figure 9. Moreover, 100 samples were acquired from different parts of both the result files (approximately 140,000 lines) to calculate the average time difference (*i.e.*, the average time added by the encryption mechanism).

**Figure 10:**
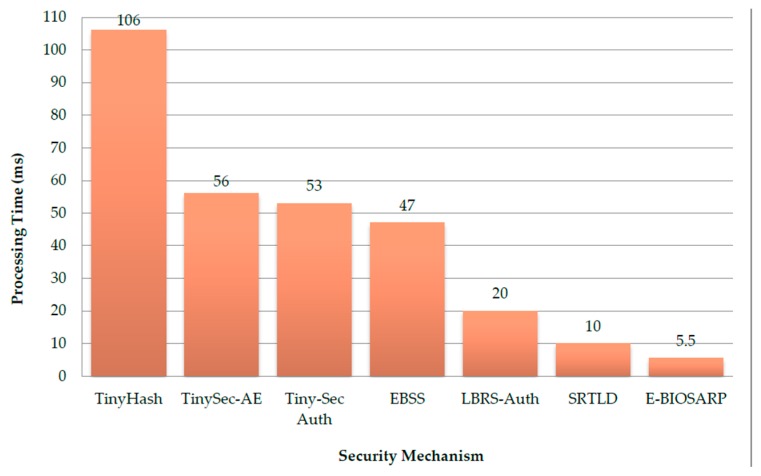
A comparison of the processing time for E-BIOSARP with those for other state-of-the-art security mechanisms: TinyHash, TinySec-AE, Tiny-Sec Auth, EBSS, LBRS-Auth, and SRTLD.

In Figure 10, the processing time values used for TinyHash, TinySec-AE, TinySec-Auth, EBSS, and LBRS-Auth were obtained from Ahmed and Fisal [26]. The experimental results confirm the rankings obtained in Section 5. Although LBRS-Auth and EBSS were found to incur the same processing time in the previous analysis, EBSS was superior to LBRS-Auth in the experiments because EBSS uses a smaller data payload size than that of LBRS-Auth. Furthermore, the results demonstrate that the average processing time per hop incurred by the E-BIOSARP encryption mechanism is less than that of SRTLD although they have the same complexity level, as described in Section 5.

The shorter processing time per hop of E-BIOSARP is primarily attributable to the efficiency, reduced complexity and better performance provided by the enhanced ACO-based routing algorithm that assists in the cost-effective encryption process. The proposed mechanism is also beneficial for network-based applications that require long durations for the transfer of data packets from a source to a destination, *i.e.*, in which the route contains numerous hops. In Section 5, we assumed that the routing functionalities, such as packet processing, packet buffering, channel access contention, and packet transfer, take the same time for all nodes and for all types of implementation (*i.e.*, with and without a security scheme). This assumption does not hold when measuring $\Delta T_{\sec}$. The routing decision scheme adopted by E-BIOSARP, which is based on end-to-end delay and link quality, reduces packet transfer time and further reduces the security processing time per hop.

### 6.4. Delivery Ratio Comparison of E-BIOSARP, BIOSARP and SRTLD

**Figure 11:**
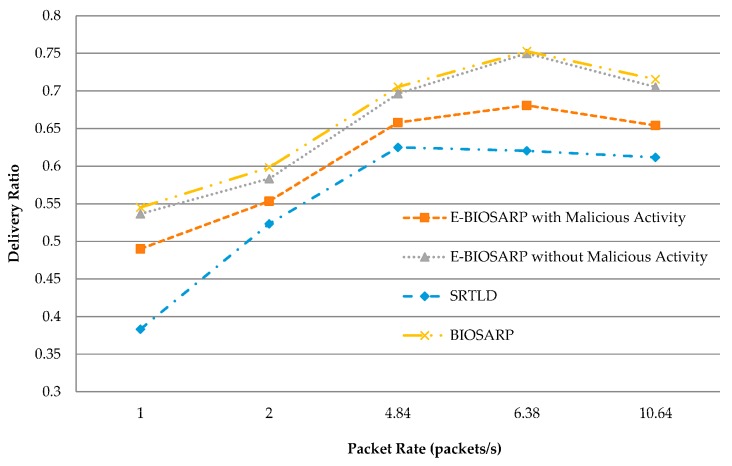
Delivery ratio comparison.

The average simulated delivery ratio results obtained for E-BIOSARP, BIOSARP and SRTLD at various packet rates for the scenario depicted in Figure 5 are summarized in Table 6. E-BIOSARP provides a higher data delivery ratio compared with the state-of-the-art SRTLD routing protocol but a lower delivery ratio than that of BIOSARP, as shown in Figure 11. The results presented in Table 6 and Figure 11 show that in the absence of malicious nodes, the delivery ratio of E-BIOSARP is superior to that of SRTLD because the processing time required by E-BIOSARP to transfer the packets is less. The advantage gained by reducing the processing time ultimately helps to decrease the delay in the secure delivery of data packets. Moreover, also in the absence of malicious nodes, the delivery ratio of E-BIOSARP is reduced only negligibly compared with that of BIOSARP as a result of the introduction of the encryption process. Furthermore, the results presented in Table 6 and Figure 11 demonstrate that when malicious traffic is present in the network, E-BIOSARP successfully handles the attacks and largely maintains its delivery ratio, with only a slight decrease due to the time required to process, classify and drop the malicious packets.

The proposed mechanism reduces the broadcast overhead and the overall processing time by avoiding repetitive rediscoveries, replies, and recalculations; this, in turn, helps to reduce processing delays and the consumption of power from the batteries of the wireless sensor nodes, thereby increasing the WSN lifetime.

### 6.5. Energy Consumption Comparison for E-BIOSARP, BIOSARP and SRTLD

**Figure 12:**
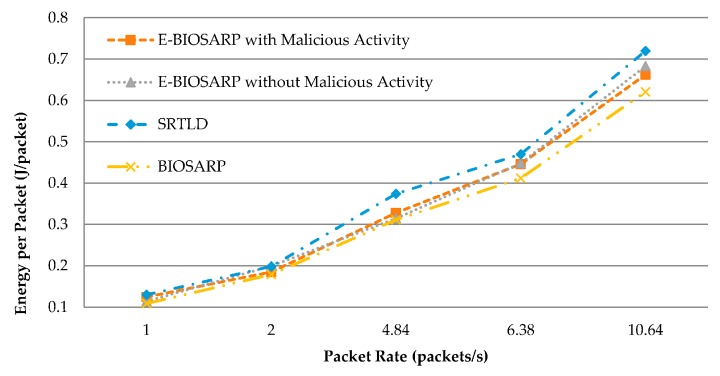
Energy consumption comparison.

The average energy consumption results for E-BIOSARP, BIOSARP, and SRTLD are compared in Table 7. Figure 12 shows that the consumption of battery power needed for SRTLD to process the packets is higher than that needed for E-BIOSARP. The latter is close to BIOSARP in terms of the energy required to process a packet. The energy consumption is reduced for E-BIOSARP compared with SRTLD because the data encryption processing time is shorter and there are fewer rediscoveries. Furthermore, Table 7 and Figure 12 show that even with the presence of malicious activity and attacks in the network, the level of energy consumption of E-BIOSARP is maintained, demonstrating the efficiency of the proposed mechanism.

### 6.6. Packet Overhead Comparison between E-BIOSARP, BIOSARP and SRTLD

**Figure 13:**
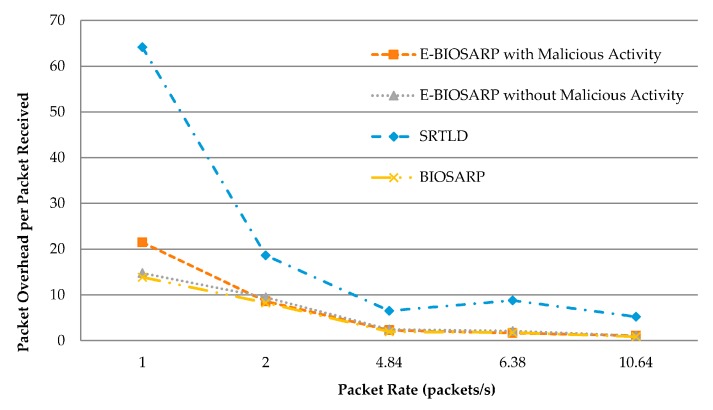
Packet overhead comparison.

The average packet overhead results for E-BIOSARP, BIOSARP, and SRTLD are compared in Table 8. Figure 13 shows the large differences between E-BIOSARP and BIOSARP on the one hand and SRTLD on the other hand. The results for E-BIOSARP are very close to the results for BIOSARP. The results for E-BIOSARP justify the reduced rediscovery and calculation process, which causes the overhead per packet received to be much less with E-BIOSARP, even when the WSN suffers from malicious activity.

### 6.7. Detection Rate and Accuracy Comparison between E-BIOSARP and SRTLD

Table 9 shows a comparison between E-BIOSARP and SRTLD in terms of the detection rate and accuracy, as determined based on the WSN scenario depicted in Figure 5. The detection rate and accuracy were calculated as described in Section 6.2.

### 6.8. Comparative Analysis of E-BIOSARP and ESPA

**Figure 14:**
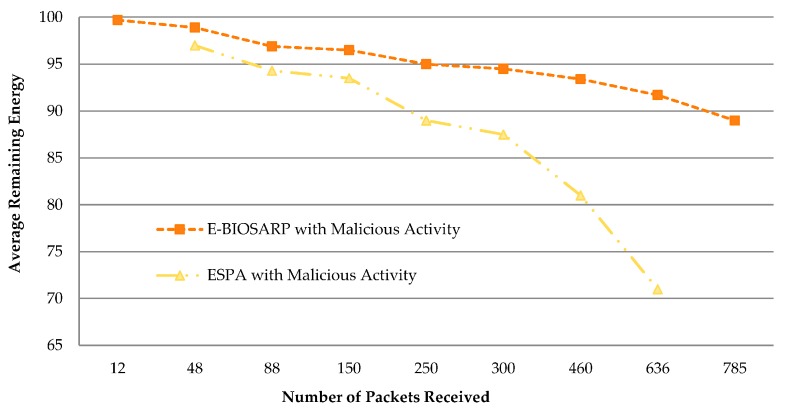
Average remaining energy.

E-BIOSARP and a recent ESPA [29] were compared in an ns-2-based simulation analysis. The network topology was defined using the simulation parameters given in [29]. ESPA exhibits much better performance than EEABR [46] and SERP [15], as presented in [29]. A WSN with 300 wireless sensor nodes was deployed in a 300 m × 300 m region, with a base station with ID 0 in the center of the network. The real-time traffic scenario was configured by using a UDP transport layer to handle the generated CBR traffic. The average difference in energy consumption between E-BIOSARP and ESPA is presented in Table 10, revealing a 7.6% increase in energy consumption when E-BIOSARP is utilized in a WSN. The advantage of E-BIOSARP based on random key encryption is illustrated in Figure 14. As the level of malicious activity increases, the performance of ESPA dramatically decreases, whereas that of E-BIOSARP remains more stable.

## 7. Concluding Remarks

In this paper, to ensure the security of WSNs beginning in the deployment phase, BIOSARP was enhanced through the incorporation an encryption mechanism that was originally applied in SRTLD to take advantage of the best features of both schemes. This approach was taken because BIOSARP achieves superior data packet routing performance compared with SRTLD, whereas the SRTLD encryption mechanism provides better communication security with minimal processing time. Under the enhanced BIOSARP (E-BIOSARP), the data to be transmitted are fragmented and only some of the important packet header fields are encrypted instead of the complete packet to save processing time and battery power. As a result, if malicious nodes and/or adversaries capture a secure packet, they cannot read the packet’s movement or its destination, which ensures network integrity and confidentiality. In addition, E-BIOSARP is capable of providing countermeasures against selective forwarding, brute-force or exhaustive key search, spoofing, eavesdropping, replaying or altering of routing information, cloning, acknowledgment spoofing, HELLO flood attacks, and Sybil attacks. The performance of E-BIOSARP was analyzed by varying the number of malicious nodes in the network. Overall, E-BIOSARP demonstrated improved performance compared with SRTLD and ESPA, and the simulation results confirm the efficiency of the proposed mechanism. Because of the encryption process, E-BIOSARP provides better security compared with BIOSARP while incurring only a minimal performance penalty because of the additional processing time required for encryption. Future work will address the enhancement of the security mechanism with additional rounds of encryption, and we will test different variations of E-BIOSARP in a real-time experimental WSN test bed for further analysis.

## Figures and Tables

**Table 1:** Comparison of recent routing protocols for WSN security.

Related Work	Attacks Covered	Shortcomings/Limitations
SERP [32]	Heavy flooding attacks	The shared secret key is pre-stored. The secret key is preloaded and is not self-maintained by the system in real time. The source node ID is readable. The system is vulnerable to brute-force search and eavesdropping attacks.
SRTLD [26]	Selective forwarding attacks Sinkhole attacks HELLO flood attacks Sybil attacks Wormhole attacks	The parameter weights for routing optimization must be re-evaluated according to location. Every sensor node in the WSN is programmed with a static location. The sink node is assumed to be a trusted base station.
ESPA [29]	Brute-force search attacks Denial of service attacks	The main commands are generated from a centralized location. Massive traffic overhead consumes an enormous amount of energy over the network. The network collapses when and where key information is not shared.
BIOSARP [23,27]	Sybil attacks Selective forwarding attacks Replayed or altered routing information attacks Acknowledgment spoofing attacks HELLO flood attacks	Not tested for unknown attacks. Needs to be tested on a real experimental WSN test bed including all attacks.
DCHM [38]	Jamming attacks Falsification of local values Falsification of fusion	The need for a trust system and feedback from the base station to the cluster heads generates enormous data packet overhead. Assumes that the base station is a trusted computing platform. The cluster key is unique for every cluster, increasing complexity. Has not been compared with any state-of-the-art security algorithm.
DWGRP [18]	Wormhole attacks	Beacon packets are broadcasted with unencrypted information. Nodes rebroadcast at an interval of 0.3 s. Very costly because the nodes require a GPS.

**Table 2:** Comparison of security schemes.

Security Scheme	Security Functions	Ranking of the Scheme
TinyHash	Encrypt(D) + MAC(D)	5
TinySec-AE	Encrypt(P) + MAC(D)	4
TinySec-Auth	MAC(D)	3
LBRS-Auth and EBSS	MAC(P)	2
SRTLD and E-BIOSARP	Encrypt(*S_ID* + *D_ID* + *Pkt_ID*)	1

**Table 3:** Network parameters used to simulate the security mechanism.

Low-rate WPAN	IEEE 802.15.4
Physical type	Phy/WirelessPhy/802_15_4
MAC type	Mac/802_15_4
Propagation model	Shadowing
Acknowledgment	Yes
Operation mode	Unslotted Non Beacon
Frequency	2.4 GHz
Initial energy	3.3 J
Power transmission	1 mW
CSThresh_	1.10765 × 10^−11^ Hz
RXThresh_	1.10765 × 10^−11^ Hz
Traffic utilized	Constant Bit Rate (CBR) with Packet Size 70 bytes
Transport layer	User Datagram Protocol (UDP)
Simulation duration	100 s

**Table 4:** Influence of malicious nodes on network performance.

Adversary Nodes	Delivery Ratio	Energy per Packet (J/Packet)
4	0.5322	0.0893563
8	0.4963	0.089909
12	0.54358	0.14805
16	0.5163	0.0891
20	0.251497	0.26612

**Table 5:** Influence of malicious nodes on network performance in terms of security.

Adversary Nodes	Detection Rate and Accuracy in Percentage	
	SRTLD	E-BIOSARP
4	6/25 = 24%	14/18 = 78%
8	20/34 = 58.82%	18/22 = 82%
12	37/45 = 82%	43/51 = 84.3%
16	64/86 = 74.42%	67/84 = 79.762%
20	498/572= 87.1%	650/662 = 98%

**Table 6:** Delivery ratio comparison.

Packet Rate (Packets/s)	E-BIOSARP without Malicious Nodes	E-BIOSARP with Malicious Nodes	BIOSARP without Malicious Nodes	SRTLD without Malicious Nodes
1	0.536667	0.49	0.5453	0.3833
2	0.58345	0.553512	0.59838	0.523411
4.84	0.6964	0.658167	0.7054	0.625
6.38	0.75	0.68094	0.7528	0.6204
10.64	0.7052	0.65424	0.7154	0.61167
**Average**	**0.6543434**	**0.6073718**	**0.663456**	**0.552756**

**Table 7:** Energy consumption comparison.

Packet Rate (Packets/s)	E-BIOSARP without Malicious Nodes	E-BIOSARP with Malicious Nodes	BIOSARP without Malicious Nodes	SRTLD without Malicious Nodes
1	0.1142	0.1245	0.1087	0.13
2	0.19953	0.185057	0.1789	0.19856
4.84	0.314607	0.32807	0.3113	0.374
6.38	0.44784	0.4458	0.41201	0.47
10.64	0.68321	0.6625	0.62102	0.72
**Average**	**0.3518774**	**0.3491854**	**0.326386**	**0.378512**

**Table 8:** Data packet overhead comparison.

Packet Rate (Packets/s)	E-BIOSARP without Malicious Nodes	E-BIOSARP with Malicious Nodes	BIOSARP without Malicious Nodes	SRTLD without Malicious Nodes
1	14.7702	21.483	13.875	64.1652
2	9.483	8.60725	8.274	18.65
4.84	2.473	2.283	1.952	6.543
6.38	2.1063	1.6626	1.7739	8.8
10.64	1.097	1.1053	0.849	5.22307
**Average**	**5.9859**	**7.02823**	**5.34478**	**20.676254**

**Table 9:** Detection rate and accuracy comparison.

Packet Rate (Packets/s)	Detection Rate and Accuracy in Percentage		
	SRTLD	E-BIOSARP	Difference
1	85/110 = 77.3%	109/128 = 85.2%	7.9%
2	128/161 = 79.5%	137/154 = 89%	9.5%
4.84	196/243 = 81%	134/151 = 88.74%	7.74%
6.38	187/239 = 78.24%	140/151 = 92.72%	14.48%
10.64	144/176 = 82%	411/429 = 95.8%	13.8%
Average Accuracy	79.608%	90.292%	10.684%

**Table 10:** Energy consumption comparison.

Packet Rate (Packets/s)	E-BIOSARP	ESPA	Difference
12	99.7		
48	98.9	97	1.9
88	96.9	94.3	2.6
150	96.5	93.5	3
250	95	89	6
300	94.5	87.5	7
460	93.4	81	12.4
636	91.7	71	20.7
785	89		
**Average**	****	****	**7.6571429**
